# Neurorehabilitation of social dysfunctions: a model-based neurofeedback approach for low and high-functioning autism

**DOI:** 10.3389/fneng.2014.00029

**Published:** 2014-08-07

**Authors:** Jaime A. Pineda, Elisabeth V. C. Friedrich, Kristen LaMarca

**Affiliations:** Department of Cognitive Science, University of CaliforniaSan Diego, La Jolla, CA, USA

**Keywords:** neurofeedback training, autism, low functioning autism, autism spectrum disorders, intervention studies, classical conditioning

## Abstract

Autism Spectrum Disorder (ASD) is an increasingly prevalent condition with core deficits in the social domain. Understanding its neuroetiology is critical to providing insights into the relationship between neuroanatomy, physiology and social behaviors, including imitation learning, language, empathy, theory of mind, and even self-awareness. Equally important is the need to find ways to arrest its increasing prevalence and to ameliorate its symptoms. In this review, we highlight neurofeedback studies as viable treatment options for high-functioning as well as low-functioning children with ASD. Lower-functioning groups have the greatest need for diagnosis and treatment, the greatest barrier to communication, and may experience the greatest benefit if a treatment can improve function or prevent progression of the disorder at an early stage. Therefore, we focus on neurofeedback interventions combined with other kinds of behavioral conditioning to induce neuroplastic changes that can address the full spectrum of the autism phenotype.

## Aim

In this review, we highlight preliminary yet promising observations to support the hypothesis that neurofeedback training (NFT) in combination with a new behavioral intervention, TAGteach, is a viable treatment option not only for high-functioning but also for low-functioning children with ASD.

## Symptoms

Autism Spectrum Disorder (ASD) is now estimated to affect 1 in 68 children (Baio, 2014), who show marked deficits in social and communicative skills, including imitation, empathy, and shared attention, as well as restricted interests and repetitive patterns of behaviors. These problems significantly affect social interactions and prevent children from establishing normal social relationships with others. The ASD phenotype varies in severity and character, encompassing individuals who are asocial but otherwise high-functioning (sometimes at the “savant” level), and low-functioning non-verbal individuals. The distinction between low and high-functioning autism is typically based on the child’s IQ, with the cutoff being around 80. At the high-functioning end of the spectrum, the deficits primarily impair social interactions and prevent children from establishing adequate relations with others. At the low-functioning end, children show interactions mainly with the goal of behavior regulation such as protesting rather than social engagement (Maljaars et al., 2012).

## Causes and brain correlates

Different models to explain autism have been proposed, but the neuroetiology of autism is not yet entirely understood. Rubenstein and Merzenich (2003) have proposed an “increased ratio of excitation/inhibition in sensory, mnemonic, social and emotional systems”, which can be caused by a combination of environmental and genetic factors. Another working hypothesis is that a dysfunction in motor processing, specifically in the mirror neuron system (MNS; di Pellegrino et al., 1992; Williams et al., 2001; Rizzolatti and Craighero, 2004), is an underlying factor in the condition. In one anatomical MRI study, Hadjikhani et al. (2006) examined 14 high-functioning adults with ASD and observed significant cortical thinning compared to matched control participants. These differences were found in areas considered part of the classic MNS, such as the inferior frontal gyrus (IFG), bilateral inferior parietal lobule (IPL), as well as right superior temporal sulcus (STS). These findings have been strengthened by significant correlations with autism symptoms, as diagnosed by the autism diagnostic interview-revised or ADI-R (Lord et al., 1994). Williams et al. (2006) studied 16 adolescents with ASD during finger movement imitation. Although no effects were detected in IFG, a comparison with matched typically developing (TD) children showed reduced activation for the ASD group in bilateral IPL. Dapretto et al. (2006) reported reduced activation in IFG in another fMRI study in which they tested nine boys with ASD during imitation of emotional facial expressions. Although children were able to perform the imitation task, significantly reduced activation in IFG was detected bilaterally in a comparison with controls. In contrast, Martineau et al. (2010) reported hyperactivation of the pars opercularis (belonging to the MNS) during observation of human motion in autistic subjects compared to controls. Baastiaansen et al. (2011) reported that IFG activity during the observation of facial expressions increased with age in subjects with autism, but not in controls, suggesting improved social functioning with age. In terms of functional connectivity, Villalobos et al. (2005) found reduced fMRI connectivity between primary visual cortex and bilateral IFG during visuomotor coordination in eight participants with ASD, compared to matched TD participants. Likewise, studies using resting state (rs)-magnetoencephalography (MEG) or quantitative electroencephalography (QEEG) support the notion of dysfunctional connectivity in ASD. Tsiaras et al. (2011) showed reduced interdependence strength within bilateral frontal and temporal sensors, as well as between temporal sensors and other recording sites in a group of ASD participants. Cornew et al. (2012) indicated that children with ASD exhibited regionally specific elevations in delta (1–4 Hz), theta (4–8 Hz), alpha (8–12 Hz), and high frequency (20–120 Hz) power, supporting an imbalance of neural excitation/inhibition as a neurobiological feature of ASD. Billeci et al. (2013) showed that children with ASD present several differences in power spectra, coherence, and symmetry measures compared to controls. This is true both when the signals are acquired in resting conditions—with either open or closed eyes—and when specific tasks are performed. For all these reasons it is speculated that abnormal functional connections exist that can lead to ineffective or atypical neural communication, which in turn may impede early affective, social and communicative development. This suggests that a reasonable therapeutic approach for the treatment of ASD is to normalize abnormal functional connections (Pineda et al., 2012). However, most of these findings rely on data from high-functioning individuals (Dapretto et al., 2006). As IQ correlates with brain volume (Posthuma et al., 2002; Posthuma and Polderman, 2013), brain structure (Price et al., 2013) and brain function (van den Bos et al., 2012), it has yet to be shown if a generalization of these results to the lower end of the spectrum is justified.

## Treatments

In clinical studies, the most effective type of therapy for ASD has been behavioral intervention, with an efficacy rate of approximately 48% (Lovaas, 1987; McEachin et al., 1993; Smith et al., 2009). However, behavioral therapy is time consuming and costly for such a low potential benefit, making it entirely out of reach to the majority of the affected population, especially in developing countries. Moreover, research aimed at interventions is predominantly conducted with high-functioning adults or adolescents and a review of the literature confirms that social skills training for ASD mostly involves high-functioning children (Cappadocia et al., 2012; Wainer and Ingersoll, 2013). However, it is the younger lower-functioning groups that have the greatest need for diagnosis and treatment, who have the greatest barrier to communication (and hence the greatest need for replacement or enhancement of communication), and who may experience the greatest benefit if such tools can improve function or prevent progression of the disorder at an early stage. Ben et al. (2008, 2011) reported better outcome in behavioral therapy for children with an IQ above 70 and less severe symptoms than for children with an IQ below 70 and more deficits. This emphasizes that different treatment approaches are needed and should be used for lower functioning children on the spectrum. Thus, alternative interventions that normalize social behavior would be beneficial and warrant serious consideration.

One alternative to behavioral therapy is NFT. NFT allows for visualization of brain activity to be fed back to a user by means of a computer in a closed “neurofeedback” loop, allowing subjects to learn to control the natural operation of brain rhythms *in vivo* and in near real time (Nowlis and Kamiya, 1970; Delorme and Makeig, 2004; Delorme et al., 2011). Brain electrical rhythms are instantiated across different spatial scales (Buzsáki and Draguhn, 2004) from single neurons (Hutcheon and Yarom, 2000), to neuronal circuits (Whittington et al., 1997), to re-entrant thalamocortical and large-scale cortico-cortical networks (Lorincz et al., 2009). It is assumed that these rhythms enable the dynamic routing and gating of information via the synchronization of various bits of information (Salinas and Sejnowski, 2001; Schoffelen et al., 2005; Jensen and Mazaheri, 2010). From a systems level perspective, electroencephalographic (EEG) responses to sensory stimuli can partially be explained by transient, stimulus-induced adjustment in the phase of ongoing rhythms via phase-resetting (Sayers et al., 1974; Makeig et al., 2002; Klimesch et al., 2007). The possibility of self-directed modulation of these rhythms and phase adjustments raises an interesting set of questions. Is it possible to promote/enhance or inhibit/suppress rhythmic oscillations in distinct neural networks *in vivo*? Can we modulate these rhythms volitionally through some periodic internal input or drive? Theoretically, a functional impact is possible through the modulation of brain rhythms that may play a causal role in specific cognitive functioning.

There is consensus that EEG activity recorded on the scalp arises mainly from cortex (Pantev et al., 1991; Llinás and Ribary, 1993). Neurons firing in synchrony while an individual is at rest produce large amplitude oscillations detected over various brain regions (Hari et al., 1997; Klimesch, 1997; Pfurtscheller et al., 1997, 2000; Muthukumaraswamy and Johnson, 2004; Muthukumaraswamy et al., 2004). The mu rhythm is such an oscillation in the 8–13 Hz band, limited to brief periods of 0.5–2 s duration and recorded in the absence of movement over sensorimotor cortex. Activation by self-movement, the observation of movement, and even the imagination of movement produces desynchronization and a suppression of mu rhythm activity (Salmelin and Hari, 1994; Pfurtscheller et al., 1997). The link between mu rhythms and mirroring activity was first proposed by Altschuler et al. (1998), and thereafter by other researchers (Cochin et al., 1998, 1999; Hari et al., 2000). More recent studies have found that mu rhythm is modulated by object-directed actions (Muthukumaraswamy and Johnson, 2004). Since it is generated by activity in sensorimotor areas and mirror neurons have been located primarily in premotor areas, it is hypothesized that mu rhythm indexes downstream modulation of primary sensorimotor areas by mirroring activity in frontal cortex (Muthukumaraswamy and Johnson, 2004; Muthukumaraswamy et al., 2004; Oberman et al., 2005; Pineda, 2005). Furthermore, TD individuals can learn to modulate mu rhythms via NFT (Pineda et al., 2000, 2003), and while normal individuals exhibit mu suppression to both self-directed and observed movement, high-functioning ASD individuals fail to exhibit mu suppression to observed movement (Oberman et al., 2005).

Most NFT approaches use a simple visual stimulus or game to train individuals to increase/decrease a particular bandwidth of the EEG signal. With training, the majority of individuals develop a high level of conscious and unconscious control over their brain activity. During training, subjects are exposed to the same visual/auditory feedback or reward stimuli, and hence the entrained EEG differences most likely represent the modulation of some internal brain state associated with the event rather than to external factors. While the precise mechanisms of how using neurofeedback can induce changes in the brain are unclear, the evidence suggests they capitalize on the innate plasticity of the brain to produce neural, functional, and ultimately behavioral changes. Furthermore, the use of QEEG (Cantor and Chabot, 2009; Coben and Myers, 2010; Thompson et al., 2010a,b) combined with specific and individualized protocols (e.g., amplitude and coherence training) can help fit the training to the heterogeneity of autistic symptomatology.

The majority of studies using neurofeedback for ASD includes children at the higher end of the autism spectrum and these studies have reported significant normalization of brain functioning as well as improvement in behavior and cognitive function (Pineda et al., 2008; Kouijzer et al., 2009; Coben et al., 2010). However, the generalization of these findings to adults or very young and lower functioning children is not yet well examined (Coben et al., 2010). One concern is whether nonverbal children even understand instructions and can process the meaning of feedback. High functioning individuals seem to process external, concrete feedback in a way similar to their TD peers (Larson et al., 2012). However, a recent study suggested that nonverbal low functioning individuals with ASD are able to attribute goals to other persons but are not able to consider the person’s circumstances or the context to interpret the actions and attribute intentions as most typical developing children do (Somogyi et al., 2013). Indeed, neurofeedback-based learning is correlated with IQ (van den Bos et al., 2012).

## Treatment for low-functioning children with autism: teaching with acoustical guidance and NFT

Operant and classical conditioning principles have been key to the behavioral treatment of autism for decades (Helm, 1976; Neuringer, 2002; Pickett et al., 2009). Secondary or conditioned reinforcers, such as auditory markers, have been shown to enhance learning, including the learning of complex sequences of behaviors, through the value acquired from their associations with primary reinforcers. As lower functioning children with ASD have a greater degree of social, language, and other behavioral deficits, they have less available treatment options and tend to be precluded from research. An auditory marker appears well suited for the enhancement of skill-teaching in this population in that it eliminates the social features of verbal praise and is more precise than verbal reinforcement. Teaching with Acoustic Guidance or TAGteach,^1^ a new behavioral intervention that uses a conditioned auditory reinforcer to mark and shape behaviors in successive approximations, was recently used to accelerate learning in a child with ASD who had difficulty learning through a traditional behavioral approach (Persicke et al., 2014). Case reports on the TAGteach webpage^2^ and several case presentations at the Applied Behavioral Analysis International (ABAI) meeting have also reported that TAGteach can enhance skill acquisition rates in children with ASD who have more difficulty learning through traditional means.

Ueda (2007) demonstrated that TAGteach was effective in easing the difficulties with transitions between tasks experienced by a 3 year old with autism. After implementing TAG methodology to increase compliance with transitions, the amount of time and number of prompts decreased compared to baseline data. In a second case study of an 8 year old female diagnosed with autism, Ueda showed that TAG decreased the number of prompts and increased the level of independence in fine motor imitation skills and requesting behaviors. Also Hanson and Madden (2007) demonstrated an increase in acquisition rates for the mastery of several different target behaviors in 5 case studies with the use of TAGteach compared to the traditional operant strategies that were used in collecting baseline data. Rosenblum (2007) used TAG to teach typing to a 9-year-old male student with autism. The subject learned to type words via TAGteach with 62.3% fewer prompts than words for which TAGteach was not used. Also, Gutierrez (2007) investigated the effects of TAG on acquisition rates of imitation behaviors in two male children with autism, ages 2 and 3, following little or no progress during baseline conditions that used traditional discrete trial methods. Following 7 months of baseline data with no progress, the 3-year-old student showed 90% accuracy for two imitation responses in just 15 sessions once TAG was used. The 2-year-old student was able to acquire target behaviors in less than four TAGteach sessions following a baseline of eight sessions without TAG that showed minimal acquisition. Winkle (2007) used TAGteach with a 12 year old male diagnosed with autism to teach a social interaction by approaching a peer, getting the peer’s attention, making eye contact, and saying hello. She showed that the time for the participant to perform a behavior and the average number of cues per interaction decreased while the average number of eye contact occurrences per session increased when using TAGteach. Winkle also noted improvement in the participant’s affect and communication skills. Though preliminary, the aforementioned case studies, highlight the potential of TAGteach to enhance shaping procedures in children with ASD that have difficulty learning behaviors through traditional behavioral methods could prove to be highly valuable to the field.

There is a need for the empirical exploration of novel treatments that target the core deficits of autism, align themselves with prominent neuroetiological theories, and are suitable for lower functioning populations. To that end, we have combined TAGteach with NFT to bridge the gap that currently exists and completed a case series of seven children with autism and with impairments that would typically preclude them from research participation in the typical study with high functioning children. The study aimed to investigate (a) whether TAGteach could be used to behaviorally prepare children to perform the skills required of an NFT intervention and EEG outcome tasks; and (b) whether electrophysiological changes or improvements in behavioral outcomes would occur in participants learning to self-regulate mu rhythms through operant strategies.

Participants were required to have a diagnosis of Autistic Disorder, be between the ages of 8 and 12, and have an IQ below 80. TAGteach was first used to shape a set of prerequisite behaviors in all participants so they could undergo an electrophysiological assessment, namely the Mu Suppression Index (MSI) and NFT thereafter. Mu suppression indices were calculated as the ratio of power during biological, goal-directed, and social action conditions relative to the power during the non-biological action condition, which is a type of movement that does not typically produce mu rhythm suppression. Following completion of pre-test measures, participants attended NFT sessions for 30–45 min 2 or 3 times per week, totaling 20 h over 20 weeks. They were trained to control power in the mu band (8–13 Hz over electrode site C4) as well as a second frequency range (43–59 Hz) recorded over C4 that reflects muscle movement artifact. A secondary auditory reinforcer, used in the TAGteach part of the study, continued to mark when participants exceeded the mu power threshold during NFT. This was followed by the primary reinforcer, which included either a videogame or preferred DVD movie that played upon mu activity exceeding a threshold. Thresholds were set based on initial individual assessment and increased when participants showed learning. Following the completion of treatment, post assessments were completed by participant and caregiver.

**Figure 1:**
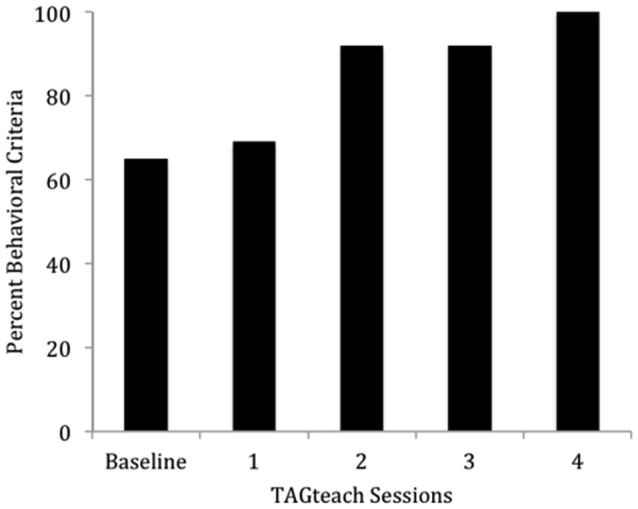
**Percent of behavioral criteria required to participate in neurofeedback training met by Case 2 across baseline and four TAGteach sessions**. Cases 1–6 met 100% criteria in less than 6.25 h (*M* = 4.71, *SD* = 1.16) of training.

**Figure 2:**
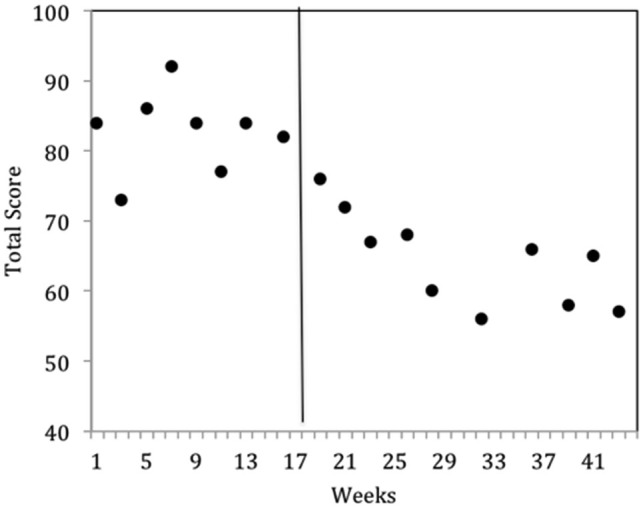
**Behavioral improvements of Case 2 at onset of neurofeedback training indicated by parent questionnaire, the Autism Treatment Evaluation Checklist (ATEC)**. ATEC Total scores across baseline (weeks 1–17) and intervention (weeks 18–43) phases indicated a significant improvement in mean scores. Lower ATEC scores indicate lesser severity.

The results were highly encouraging in that all participants learned to perform the behaviors required of NFT and EEG outcome tasks in an average of 5 h over 6 TAGteach sessions (see Figure 1). The administration of NFT was entirely feasible via TAGteach in these children, providing a promising way to normalize mu suppression responses and improve behavior (see Figure 2). Furthermore, the results suggested that biofeedback-assisted TAGteach training could indeed help children with low functioning autism to better sustain complex skills and reduce artifact-creating behaviors for the required durations. These findings support TAGteach as a feasible method of preparing lower functioning children on the spectrum to cooperate with research tasks and participate in an otherwise inaccessible, or difficult to implement, treatment.

## Conclusion

There are few research studies including children on the lower end of the autism spectrum, which creates poor treatment options for this population. In this review we present TAGteach in combination with NFT as a promising alternative that may be suitable for low functioning children on the spectrum.

## Conflict of interest statement

The authors declare that the research was conducted in the absence of any commercial or financial relationships that could be construed as a potential conflict of interest.

## Footnotes

^1^http://www.TAGteach.com

^2^http://www.TAGteach.com/Autism_And_Special_Education
